# Significance of KRAS/PAK1/Crk pathway in non-small cell lung cancer oncogenesis

**DOI:** 10.1186/s12885-015-1360-4

**Published:** 2015-05-09

**Authors:** Fariborz Mortazavi, Jie Lu, Ryan Phan, Michael Lewis, Kenny Trinidad, Amir Aljilani, Gholamhossein Pezeshkpour, Fuyuhiko Tamanoi

**Affiliations:** 1 Division of Hematology/Oncology, West Los Angeles VA, Los Angeles, CA USA; 2 Department of Medicine, University of California, Los Angeles, CA USA; 3 Jonsson Comprehensive Cancer Center, Los Angeles, CA USA; 4 Department of Microbiology Immunology & Molecular Genetics, University of California|, Los Angeles, CA USA; 5 Department of Pathology, West Los Angeles VA, Los Angeles, CA USA

**Keywords:** KRAS, PAK1 kinase, c-Crk, E-cadherin, Cell adhesion, Lung cancer, Signal transduction

## Abstract

**Background:**

Key effector(s) of mutated KRAS in lung cancer progression and metastasis are unknown. Here we investigated the role of PAK1/Crk axis in transduction of the oncogenic KRAS signal in non-small cell lung cancer (NSCLC).

**Methods:**

We used NSCLC clinical specimens to examine the correlation among *KRAS* mutations (codon 12, 13 and 61); PAK1/Crk axis activation [p-PAK1(Thr423), p-Crk(Ser41)]; and adhesion molecules expression by immunohistochemistry. For assessing the role of proto-oncogene c-Crk as a KRAS effector, we inhibited KRAS in NSCLC cells by a combination of farnesyltransferase inhibitor (FTI) and geranylgeranyltransferase inhibitor (GGTI) and measured p-Crk-II(Ser41) by western blotting. Finally, we disrupted the signaling network downstream of KRAS by blocking KRAS/PAK1/Crk axis with PAK1 inhibitors (i.e., IPA-3, FRAX597 or FRAX1036) along with partial inhibition of all other KRAS effectors by prenylation inhibitors (FTI + GGTI) and examined the motility, morphology and proliferation of the NSCLC cells.

**Results:**

Immunohistochemical analysis demonstrated an inverse correlation between PAK1/Crk phosphorylation and E-cadherin/p120-catenin expression. Furthermore, *KRAS* mutant tumors expressed higher p-PAK1(Thr423) compared to *KRAS* wild type. KRAS prenylation inhibition by (FTI + GGTI) completely dephosphorylated proto-oncogene c-Crk on Serine 41 while Crk phosphorylation did not change by individual prenylation inhibitors or diluent. Combination of PAK1 inhibition and partial inhibition of all other KRAS effectors by (FTI + GGTI) dramatically altered morphology, motility and proliferation of H157 and A549 cells.

**Conclusions:**

Our data provide evidence that proto-oncogene c-Crk is operative downstream of KRAS in NSCLC. Previously we demonstrated that Crk receives oncogenic signals from PAK1. These data in conjunction with the work of others that have specified the role of PAK1 in transduction of KRAS signal bring forward the importance of KRAS/PAK1/Crk axis as a prominent pathway in the oncogenesis of *KRAS* mutant lung cancer.

**Electronic supplementary material:**

The online version of this article (doi:10.1186/s12885-015-1360-4) contains supplementary material, which is available to authorized users.

## Background

*KRAS* mutant lung cancer comprises 25-30% of lung adenocarcinomas and unfortunately no effective treatment is currently available for this sub-type of non-small cell lung cancer (NSCLC). One strategy to interrupt the oncogenic KRAS signal is to block the key downstream effector(s) of this oncogene. Recently, PAK1 kinase was shown to play a role in transduction of the KRAS signal [1-4]. For example, exposure of cells that harbor *KRAS* or *NRAS* mutations to PAK1 inhibitor (IPA-3) resulted in cell death while this inhibitor had no effect on *BRAF* mutant cells [3]. Furthermore, knockdown of PAK1 in *KRAS* mutant colon cancer cells inhibited the proliferation of these cells independent of Raf/MEK/ERK or PI3K/Akt pathways [4]. Our data previously showed that PAK1 phosphorylates adaptor protein Crk and thereby promotes cell motility and cell invasiveness [5]. Considering Crk can function as an onco-protein [6-8], we hypothesized that KRAS/PAK1/Crk axis plays a prominent role in transduction of oncogenic KRAS signal. Here, we demonstrate that inhibition of KRAS/PAK1/Crk pathway in conjunction with partial widespread interruption of KRAS signal dramatically alters the morphology, motility and proliferation of *KRAS* mutant NSCLC cells.

## Methods

### Cell cultures

H157 and Rh2 cells were routinely cultured in RPMI supplemented with antibiotics and 10% heat-inactivated FBS (Omega Scientific, Tarzana, CA) along with Penicillin-Streptomycin (Life Technologies, Grand Island, NY Cat. number 15140-122) without any additional L-glutamine.

### Western blots

NSCLC cell lines were seeded in 10 cm Petri dishes at 5 x 10^5^ cells per dish, which resulted in 30-40% confluency 24 hours after plating. Cells were harvested at 24 hours by adding trypsin, pelleted and lysed in 100 μl of lysis buffer (NaCl 15 mM; EDTA 0.5 mM; Tris 10 mM) using a Branson Sonifier. Cell debris was collected by centrifugation at 4°C, and protein concentration was measured by the BCA method. Protein was resolved by SDS-PAGE and was transferred to a nitrocellulose membrane. The membrane was blocked with TBS with 5% nonfat powdered milk.

Membranes were immunoblotted with the following primary antibodies: PAK1 (Sigma-Aldrich Cat. number SAB4300427; 1:1000), p-Thr 423 PAK1 (Cell signaling Cat. Number 2601; 1:1000); E-cadherin (BD biosciences Cat. number 610181; 1:10,000); p120 catenin (BD biosciences Cat. number 610133; 1:4000); Crk-II (Santa Cruz Biotechnology Cat. number sc-289; 1:200); p-Ser41 Crk-II (Santa Cruz Biotechnology Cat. number sc-130186; 1:100).

Horse radish peroxidase conjugated secondary antibodies were used for detection of bands by chemiluminescence (ECL western blotting detection reagents, Amersham Biosciences, Piscataway, NJ, USA).

### Immunohistochemical stating and determination of intensity of staining

Paraffin embedded NSCLC clinical specimens from surgically resected specimens at the West Los Angeles Veterans Administration were selected. Specimens were formalin fixed, processed and sectioned at 4 μm. The glass slides were deparaffinized and stained by DAKO AutostainerLink48 by the following primary antibodies: PAK1 (Sigma-Aldrich Cat. number SAB4300427); p-Thr 423 PAK1 (Cell signaling Cat. Number 2601); E-Cadherin (BD biosciences Cat. number 610181); p120 Catenin (BD biosciences Cat. number 610133); Crk-II (Santa Cruz Biotechnology Cat. number sc-289); p-Ser41 Crk-II (Santa Cruz Biotechnology Cat. number sc-130186). Following tissue staining, the slides were reviewed by two pathologists and the intensity of staining in each slide was ranked according to a scale from (0 to 3+; No staining was designated as 0 and strongest staining for each antibody as 3+).

### *KRAS* mutation analysis

The status of *KRAS* mutation on codon 12, 13 and 61 was examined by sequencing the *KRAS* exons 2 and 3. Initially, an H&E staining from each tumor specimen was obtained and reviewed to accurately select tumor area. Three to five adjacent unstained slides of 5-7 μm was obtained from the corresponding paraffin-embedded (FFPE) block and the tumor containing areas was harvested for extraction of genomic DNA. DNA was extracted and purified by using Qiagen kit (Life Technologies) according to the manufacturer’s instructions and DNA quality and quantity was measured and recorded. Subsequently, we proceeded to *KRAS* mutation analysis. We have recently developed a real-time PCR-based approach to rapidly screen for mutation of various genes including *KRAS* by using in-house developed Taqman probes that specifically recognize wild type and mutant alleles of each gene. This methodology has proven to be highly specific and sensitive and applicable to various sample types including formalin-fixed paraffin embedded (FFPE) tissue. In this study, *KRAS* mutation analysis was performed for specific 12 known codon 12 and codon 13 mutations. For codon 61 mutations, we sequenced *KRAS* exon 3.

### Wound healing assays and microscopy

A549 and H157 cells were plated in a 6 well plate dish at 1 x 10^5^ cells per well and were grown to confluent stage. By using a sterile P1000 pipette tip, a straight scratch was made along the largest diameter of each well and a baseline photomicrograph was taken from this scratch with two different magnifications. A follow up photomicrograph was taken at 24 hours. Photomicrographs of the cells were obtained by a Nikon Eclipse TS100 inverted microscope equipped with a Cannon A510 digital camera. The digital camera was connected with a Max View Plus adaptor (ScopeTronix) to the inverted microscope. An equal area of the photomicrographs from each condition was imported into the Adobe Photoshop software. The wound area was selected by using the Magic Wand Tool of the Adobe Photoshop and the number of pixels in each selected area was determined by the Histogram Tool. Experiments were repeated three times and the average wound surface area was compared among the groups.

### Determining cell proliferation

Cells were seeded in 24 well plates and exposed to inhibitors [IPA-3 (Tocris Biosciences, Cat. number 3622); FRAX597 and FRAX1036 (Genentech)] at desired concentration. Cells from each well were harvested in triplicates daily and counted by hemocytometer. The mean of cell counts were plotted over the course of five days.

### Ethics

Human subject specimens were deidentified prior to use in this project. The utilized method was reviewed and approved by the West Los Angeles Veterans Administration Institutional Review Board.

## Results

### Phosphorylation of PAK1/Crk is inversely correlated with E-cadherin/p120-catenin expression in clinical NSCLC specimens

Our previous work showed that PAK1 phosphorylates adaptor protein Crk on serine 41 which in turn increases motility and invasiveness of lung cancer cells [5]. Furthermore, we have shown that adaptor protein Crk transcriptionally represses *(CTNND1) p120-catenin* promoter [9] while loss of p120-catenin has been shown to result in E-cadherin degradation and destabilization of adherens junctions [10-12]. Here we sought to investigate whether PAK1, Crk, p120-catenin and E-cadherin establish a correlation with each other in clinical lung cancer specimens. For this purpose, we examined the expression of PAK1, p-PAK1(Thr423), Crk-II, p-Crk-II(Ser41), p120-catenin and E-cadherin in surgically resected paraffin embedded NSCLC specimens.

**Figure 1:**
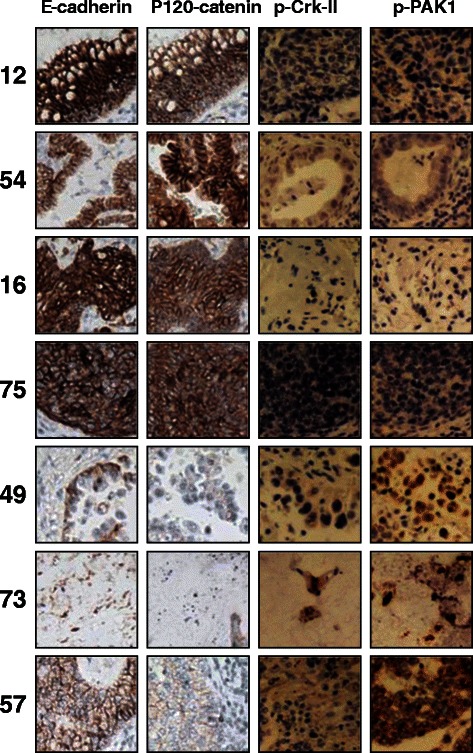
PAK1 activation and Crk phosphorylation are correlated with loss of E-cadherin and p120-catenin in NSCLC specimens. Photomicrographs demonstrating immunohistochemical staining of NSCLC clinical specimens. Samples 12, 54, 16 and 75 harbor high E-cadherin/p120-catenin while expressing no detectable level of p-PAK1(Thr423) and p-Crk-II(Ser41). On the other hand, samples 49, 73 and 57 with detectable p-PAK1(Thr423) and p-Crk-II(Ser41) show very low levels of E-cadherin/p120-catenin.

**Figure 2:**
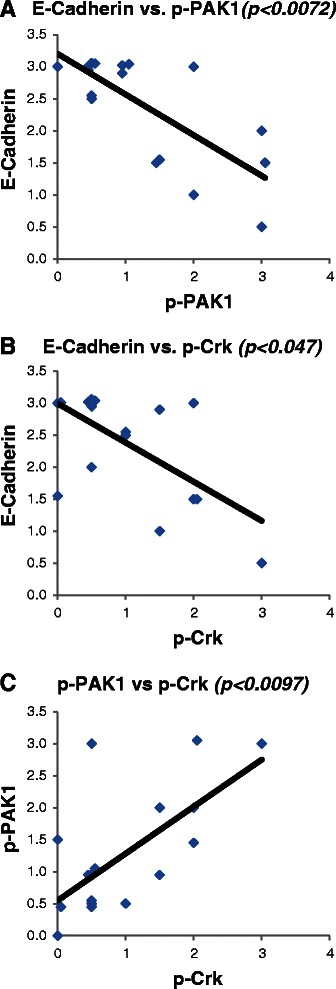
PAK1 activation and Crk phosphorylation are positively correlated while establish a negative correlation with E-cadherin in NSCLC. Dot plots demonstrating the correlation between **A**-E-cadherin and p-PAK1(Thr423); **B**-E-cadherin and p-Crk-II(Ser41); **C**-p-PAK1(Thr423) and p-Crk-II(Ser41) expression by immunohistochemistry in 17 NSCLC clinical specimens. The average intensity of protein expression across the slide is quantified in a scale from 0-3+. The correlation between the expressions of each two marker was examined by utilizing Spearman Rank Correlation statistical test.

We observed a concordance between expression of p-PAK1(Thr423) and p-Crk-II(Ser41) (Figure 1). Interestingly, tumors expressing phosphorylated PAK1/Crk had very low level of p120-catenin and E-cadherin (Figure 1). Of note, we did not observe an association between total PAK1 and Crk-II expression with that of p120-catenin/E-cadherin (data not shown). In order to examine the statistical correlation between the intensity of PAK1/Crk phosphorylation with E-cadherin expression, we quantified the phosphorylation and expression of these proteins in a scale of 1-3 as described in the Methods section. Subsequently, the correlation between the intensity of p-PAK1(Thr423), p-Crk-II(Ser41) and E-cadherin was examined in a Spearman Rank Correlation analysis (Figure 2). E-cadherin expression in the examined samples showed a statistically significant negative correlation with the expression of p-PAK1(Thr423) and p-Crk-II(Ser41) (p < 0.0072 and p < 0.047 respectively) while p-PAK1(Thr423) and p-Crk-II(Ser41) established a positive correlation with each other (p < 0.0097). These findings highly suggest that PAK1 activation by upstream stimuli results in Crk mediated suppression of p120-catenin and E-cadherin in lung cancer.

**Figure 3:**
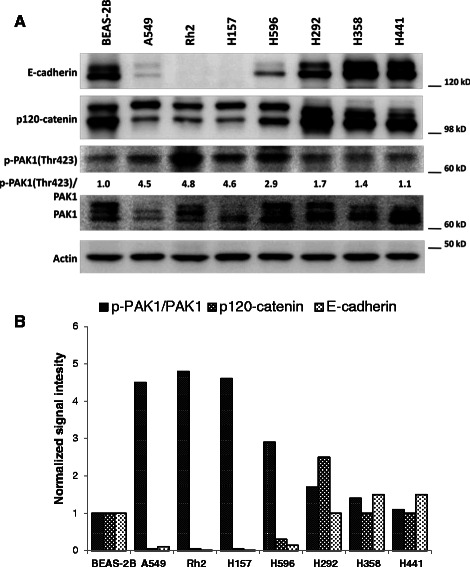
PAK1 activation is inversely correlated with E-cadherin and p120-catenin expression in NSCLC cells. **A**-Western blots demonstrating E-cadherin, p120-catenin, PAK1 and p-PAK1(Thr423) expression in a panel of NSCLC cells as well as immortalized normal human respiratory epithelial cells (BEAS-2B). **B**-Bar chart demonstrating signal intensity of p-PAK1/PAK1, p120-catenin and E-cadherin normalized to the value of BEAS-2B cells.

Furthermore, we examined PAK1 activation and its correlation with E-cadherin/p120-catenin expression in a panel of NSCLC cell lines (Figure 3). For this purpose, we examined the expression of E-cadherin, p120-catenin, total PAK1 and p-PAK1(Thr423) in a panel of NSCLC cells. As expected, we observed an inverse correlation between E-cadherin/p120-catenin expression and the ratio of phospho-PAK1/total PAK1 in the examined panel of cell lines.

### PAK1 activation is correlated with surgical stage at presentation in NSCLC

**Figure 4:**
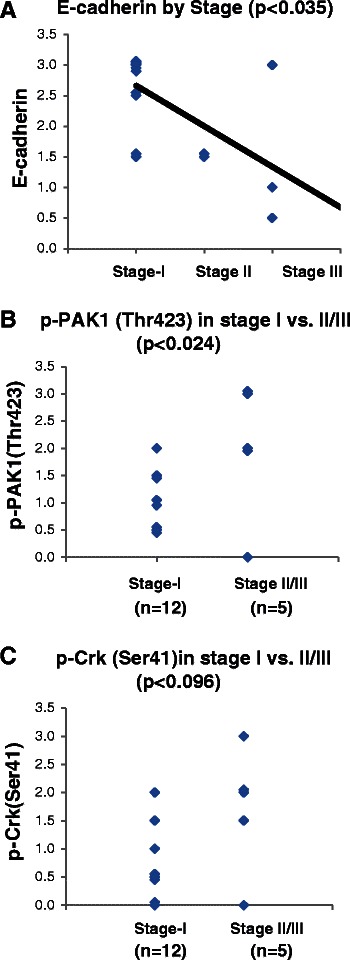
PAK1 activation is correlated with tumor stage at presentation. **A**-Dot plot demonstrating the expression of E-cadherin in the examined tumors in relation to the surgical stage of each tumor. The correlation between variables were examined by Spearman Rank Correlation analysis. **B**- Dot plot demonstrating the expression of p-PAK1(Thr423) in stage I and stage II/III tumors. The mean between groups was compared by student T-test. **C**- Dot plot demonstrating the expression of p-Crk-II(Ser41) in stage I and stage II/III tumors. The mean between groups was compared by student T-test.

Considering we identified that PAK1 activation is closely correlated with E-cadherin and p120-catenin expression in our clinical samples, we were facing the question whether PAK1 activation establishes any association with tumor stage at presentation. In order to answer this question, we reviewed the medical records for each case and extracted relevant clinical parameters to surgical staging (i.e., tumor size and mediastinal nodal involvement) and correlated the results with E-cadherin, p-PAK1(Thr423) and p-Crk-II(Ser41) expression in our samples. All examined tumors were surgically removed so none of the cases had distant metastasis therefore our cases ranged from Stage I to III. Of note, majority of resected lung tumors including cases in our repository are from stage I disease with a limited number of stage II-III cases. As expected the intensity of E-cadherin expression established a close correlation with surgical stage of tumors (Figure 4A). Furthermore, we observed that the mean of p-PAK1(Thr423) expression was significantly higher in stage II/III vs. stage I tumors (Figure 4B). However, the difference between mean of p-Crk-II(Ser41) expression in stage I vs stage II/III tumors did not reach the statistical threshold (Figure 4C). We should mention that the tumor stage (i.e., tumor size, nodal involvement and distant metastasis) is a function of (i) tumor biology, and (ii) the time interval that tumor has been growing. In other words, aggressive tumors generally present at higher stages (as we see in case of most tumors with activated PAK1) however we also see one case with no PAK1 activation and high stage. The high stage of this case can be explained by the long period of time this tumor has been growing prior to being detected and surgically removed.

### PAK1 is phosphorylated in *KRAS* mutant NSCLC specimens

**Figure 5:**
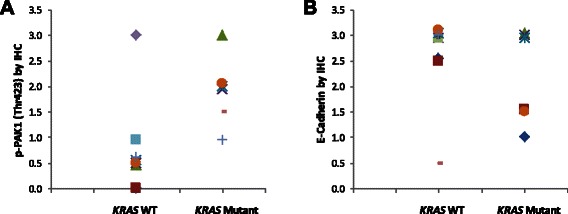
*KRAS* mutant NSCLC specimens express higher activated PAK1 compared to *KRAS* wild type samples. **A**-Dot plot demonstrating immunohistochemical expression of p-PAK1(Thr423) in *KRAS* mutant and *KRAS* wild type NSCLC clinical specimens. p-PAK1(Thr423) is detectable in all *KRAS* mutant specimens while most *KRAS* wild type samples express lower p-PAK1(Thr423). **B**- Dot plot demonstrating immunohistochemical expression of E-cadherin in *KRAS* mutant vs. *KRAS* wild type NSCLC clinical specimens.

Mutated KRAS transduces its oncogenic signal through several downstream effectors. Recently, PAK1 has been identified as one of the prominent players that transduce the oncogenic KRAS signal [1-4]. Here we examined whether PAK1 is activated in *KRAS* mutant NSCLC specimens. Towards this end, we examined the presence of *KRAS* mutations in codon 12, 13 and 61 by examining *KRAS* exons 2 and 3 (as explained in the Methods) and compared PAK1 phosphorylation between *KRAS* mutant and wild type groups (Figure 5). We observed that *KRAS* mutant group demonstrated a statistically significant higher mean of p-PAK1(Thr423) expression as compared to samples harboring wild type *KRAS* (P < 0.0265) “see Additional file 1”. Furthermore, we did not observe any *KRAS* mutant sample that did not express p-PAK1(Thr423). We should mention that we noticed one case that carried wild type *KRAS* along with elevated p-PAK1(Thr423). This finding suggests that upstream oncogenes other than mutated KRAS also transduce their signal through PAK1 (e.g., PI3K) [13]. *KRAS* mutant samples also demonstrated lower mean of E-cadherin expression compared to wild type group however this difference was not statistically significant. These data further suggest that PAK1 is activated by mutated KRAS in lung cancer.

### KRAS prenylation inhibition dephosphorylates Crk-II on Serine 41

**Figure 6:**
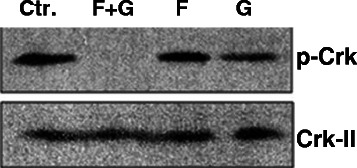
KRAS inhibition dephosphorylates Crk-II on serine 41. Western blots demonstrating loss of Crk-II phosphorylation on serine 41 in Rh2 NSCLC cells following 24 hour exposure of cells to combination of (F) farnesyltransferase inhibitor (BMS-225975 at 2 μM) and (G) geranylgeranyltransferase inhibitor (P61A6 at 2 μM).

We previously showed that PAK1 phosphorylates Crk-II on serine 41 [5] as PAK1 silencing dephosphorylated Crk-II on this residue and the amino acid sequence of Crk-II at the vicinity of Serine 41 was identical to the PAK1 phosphorylation sequence site on Raf and also this sequence showed a high degree of homology to the PAK1 phosphorylation consensus sequence in other PAK1 effectors (i.e., MEK, Snail and LIMK-1) [5,14]. Considering PAK1 is shown to be functioning downstream of KRAS, we sought to examine whether KRAS signal is transduced through Crk as well. In order to answer this question, we inhibited KRAS by a combination of farnesyltransferase inhibition (FTI) and geranylgeranyltransferase inhibition (GGTI) in NSCLC cells and examined Crk phosphorylation. Recently, we developed novel small molecule geranylgeranyltransferase type I inhibitors (GGTIs) [15-18] through a high-throughput screen. One of the identified GGTIs (i.e., P61A6) has inhibited both proliferation and cell cycle progression in a variety of human cancer cell lines including NSCLC cells [19]. Interestingly, exposure of Rh2 cells to FTI (BMS-225975) and GGTI (P61A6) combination for 24 hours completely dephosphorylated Crk-II on Serine 41 as demonstrated in a western blot assay (Figure 6). On the other hand, exposure of each inhibitor individually had little or no effect on Crk-II phosphorylation. This finding demonstrates that adaptor protein/proto-oncogene Crk is functioning downstream of KRAS. Considering (i) PAK1 is shown to be operative downstream of KRAS [1-4] and (ii) PAK1 phosphorylates Crk on serine 41 [5], it is most likely that the link from KRAS to Crk is transduced through PAK1.

### Combination of PAK1 inhibition and KRAS prenylation inhibition cause a dramatic morphological change in NSCLC cells

KRAS transduces its signal through several downstream effectors and it is likely that more than one pathway needs to be inhibited to achieve an effective KRAS signal blockade. Here, we asked whether inhibition of KRAS/PAK1/Crk axis provides adequate KRAS signal interruption when combined with inhibition of other KRAS effectors. For answering this question, we decided to inhibit PAK1/Crk axis and at the same time partially interrupt all other KRAS effectors because it is not known if any KRAS effector predominately transduces the oncogenic signal. In order to block the KRAS signal through PAK1/Crk axis, we decided to use a PAK1 inhibitor (i.e., IPA-3) while for interruption of all other KRAS effectors we used KRAS prenylation inhibitors.

Inhibitors of KRAS prenylation prevent membrane association of KRAS and thereby inhibit KRAS activation. Examples include farnesyltransferase inhibitors (FTI) and geranylgeranyltransferase inhibitors (GGTI). In order to achieve a successful loss of KRAS prenylation, a combination of FTI and GGTI is required because if FTI is used alone, geranylgeranyltransferase type I (GGTase-I) will compensate for the loss of farnesylation by enhancing geranylgeranylation of KRAS and consequently resumes membrane association and transforming activity of KRAS. We should mention that the required dose of FTI and/or GGTI that completely blocks the KRAS signal can cause loss of prenylation in other proteins thereby may result in severe cellular toxicity. Therefore, tolerable dose of (FTI + GGTI) partially blocks the KRAS signal.

**Figure 7:**
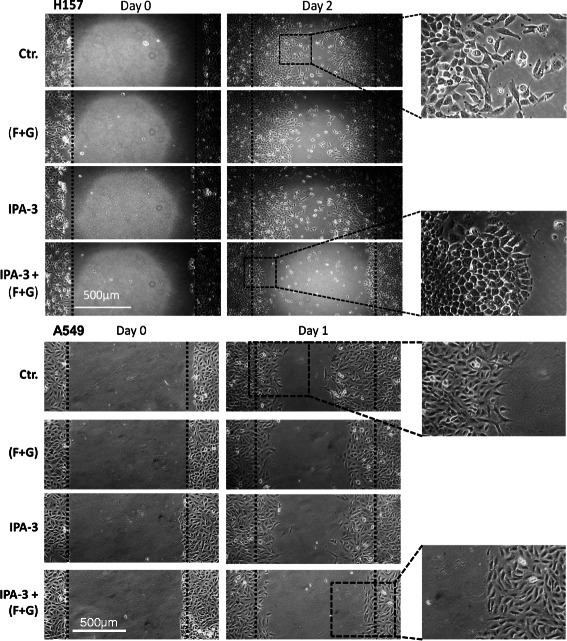
Addition of PAK1 inhibitor to KRAS prenylation inhibitors dramatically alters cell morphology and motility. Photomicrographs of standard wound healing assays in H157 and A549 cells (*KRAS* mutant). Cells were exposed to (i) farnesyltransferase inhibitor (BMS-225975 at 2 μM) and geranylgeranyltransferase inhibitor (P61A6 at 2 μM) [F + G]; (ii) PAK1 inhibitor (IPA-3 at 5 μM); (iii) combination of the three inhibitors or (iv) the inhibitor vehicles for 24-48 hours.

In fact, addition of PAK1 inhibitor (IPA-3) to (FTI + GGTI) and exposure of H157 cells to these inhibitors resulted in a prominent loss of cellular motility and a clear change in cellular morphology in a standard wound healing assay (Figure 7). H157 cells that have a mesenchymal morphology, obtained an epithelial phenotype with a smooth border when exposed to IPA-3 and (FTI + GGTI) combination for 48 hours. Of note, exposure of H157 cells to (FTI + GGTI) or IPA-3 alone did not demonstrate a noticeable effect in the wound healing assay. A similar but less pronounced effect was observed in A549 cells at 24 hours.

In order to determine whether the observed effects in wound healing assays were related to changes in cell proliferation, we examined the rate of cell proliferation as explained under the Methods section following exposure of A549 and H157 cells to IPA-3, (FTI + GGTI), or combination “see Additional file 2”. In case of H157 cells, changes in cell proliferation started after the first day of exposure to inhibitors while A549 cells showed changes in cell proliferation mainly after the second day of exposure (i.e., after completion of wound healing assay). These findings demonstrate that combination of PAK1 inhibitor and prenylation inhibitors also affect cell proliferation and therefore we conclude that the observed effect of inhibitors combination on the course of wound healing assays (Figure 7) seem to a be in part due to changes in cell motility as well as cell proliferation.

These data demonstrate that blockade of KRAS signal through PAK1/Crk axis in conjunction with a widespread partial KRAS signal interruption provide adequate disruption of signaling network (downstream of KRAS) and result in a substantial biological effect. These findings further emphasize on the role of KRAS/PAK1/Crk axis as a prominent downstream effector of KRAS in NSCLC. Furthermore, these data provide evidence that inhibition of more than one pathway is required to achieve a noticeable KRAS inhibition.

### Combination of PAK1 inhibition and KRAS prenylation inhibition dephosphorylates ERK

**Figure 8:**
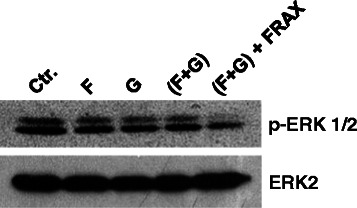
Addition of PAK1 inhibitor to KRAS prenylation inhibitors dephosphorylated ERK. Western blots demonstrate ERK dephosphorylation following 24 hour exposure of Rh2 cells to (F) farnesyltransferase inhibitor (BMS-225975 at 2 μM), (G) geranylgeranyltransferase inhibitor (P61A6 at 2 μM), combination of the two (F + G), or (F + G) along with PAK1 inhibitor (FRAX597 at 40 μM).

KRAS transduces its signals through several downstream effectors including PAK1, Raf/MEK/ERK, RalGDS, PDK1, Tiam1 and phospholipase C-epsilon (PLCξ) among others. Here we sought to investigate whether KRAS prenylation inhibition along with PAK1 inhibition might affect KRAS effectors other than Crk. For this purpose we exposed Rh2 cells to FTI (BMS-225975); GGTI (P61A6); combination of the two (FTI + GGTI); and also (FTI + GGTI) along with PAK1 inhibitor (FRAX597, Genentech). Interestingly, neither FTI/GGTI nor their combination could alter ERK phosphorylation however addition of PAK1 inhibitor (i.e., FRAX597) to (FTI + GGTI) significantly dephosphorylated ERK (Figure 8).

### Combination of PAK1 inhibition and KRAS prenylation inhibition alters proliferation of NSCLC cells

**Figure 9:**
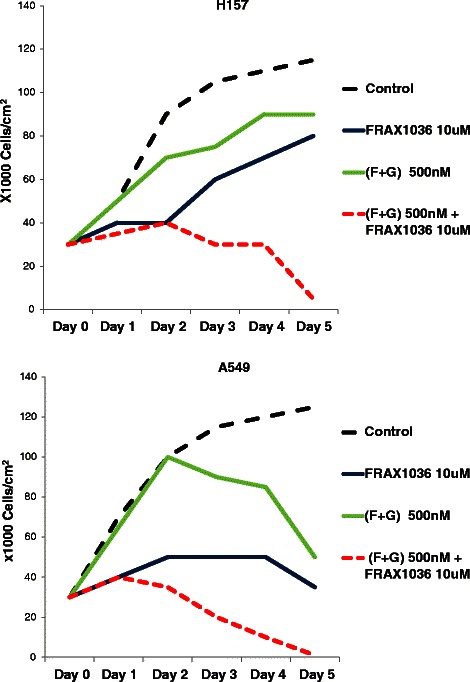
Addition of PAK1 inhibitor to KRAS prenylation inhibitors alters the proliferation of NSCLC cells. Line charts demonstrating mean cell count of H157 and A549 cells upon exposure to prenylation inhibitors [FTI (BMS-225975) and GGTI (P61A6) at 500 nM each], PAK1 inhibitor (FRAX1036 at 10 μM) or combination in comparison to cells exposed to inhibitors’ diluent (DMSO).

Considering downstream effectors of both PAK1 and KRAS include modulators of cell proliferation, we investigated whether inhibition of PAK1 along with KRAS prenylation inhibition may alter proliferation of NSCLC cells. Towards this end, we exposed A549 and H157 cells to (i) prenylation inhibitors [i.e., combination of FTI (BMS-225975) and GGTI (P61A6) at 500 nM each], (ii) PAK1 inhibitor (FRAX1036 at 10 μM, Genentech), (iii) prenylation inhibitors and PAK1 inhibitor, and (iv) inhibitors’ vehicle (Figure 9). H157 cells continued to grow in the presence of either prenylation inhibitors or PAK1 inhibitor at the above mentioned concentrations however combination of prenylation inhibitors and PAK1 inhibitor synergistically reduced the proliferation of these cells. On the other hand, proliferation of A549 cells were affected by PAK1 or prenylation inhibitors to some extent nevertheless combination of PAK1 and prenylation inhibitors showed a much stronger effect in halting the proliferation of A549 cells.

## Discussion

*KRAS* mutations comprise a large fraction of driving mutations in a variety of tumors. While this abnormality has been the focus of intense investigations, there has been little success in counteracting the oncogenic effects of KRAS. Direct KRAS targeting has proven to be difficult because several different mutations in codon 12, 13 and 61 of *KRAS* can produce a dysfunctional protein that has lost its ability to relieve from activation. Other strategies for interruption of oncogenic KRAS signals have been employed such as inhibiting post-translational modification and membrane association of Ras proteins. C-terminal farnesylation of Ras is critical for Ras localization to the plasma membrane and for Ras binding to effector molecules in the various downstream signaling pathways [20-24]. Multiple approaches have been utilized to interfere with Ras binding to the plasma membrane with limited success including (i) farnesyltransferase inhibition (FTI); (ii) inhibition of Ras-converting enzyme-1 (RCE1) and isoprenylcysteine carboxylmethyltransferase (ICMT); (iii) employment of farnesyl moiety-containing molecules, and (iv) geranylgeranyltransferase type I (GGTase-l) inhibition [25]. Several FTIs have been developed and tested in clinical settings including Tipifarnib (R115777) and Lonafarnib (SCH66336) but unfortunately clinical trials in pancreatic cancer did not show any clinical benefit of using FTIs alone. The failure of FTIs could be because of the addition of a geranylgeranyl group by GGTase-I which can effectively substitute for the farnesyl group and thereby support Ras membrane association and resume transforming activity. This notion supports strategies to combine FTI with GGTI for effective loss of KRAS prenylation. Despite the above, previous generation of GGTIs demonstrated significant toxicity when combined with FTI at the required concentration to achieve loss of KRAS prenylation [26]. We should mention that, prenylation inhibitors affect prenylation of many proteins other than KRAS therefore only a limited dose of these inhibitors can be tolerated and the tolerable dose of (FTI + GGTI) combination most likely provides a partial blockade of the oncogenic KRAS signal.

Blocking the downstream KRAS effectors has also come forward as a promising strategy to block the oncogenic KRAS signal. Several downstream signaling pathways transduce KRAS signal including PAK1, Raf/MEK/ERK, RalGDS, PDK1, Tiam1 and phospholipase C-epsilon (PLCξ) among others however it is not clear if any of these pathways exclusively transduce the oncogenic KRAS signal. Most likely, each downstream pathway participates in one or more aspects of KRAS mediated oncogenesis (e.g., proliferation, invasion/metastasis, apoptosis resistance, etc.). Therefore, an effective blockade of the oncogenic KRAS signal most likely requires signal inhibition in more than one downstream pathway.

**Figure 10:**
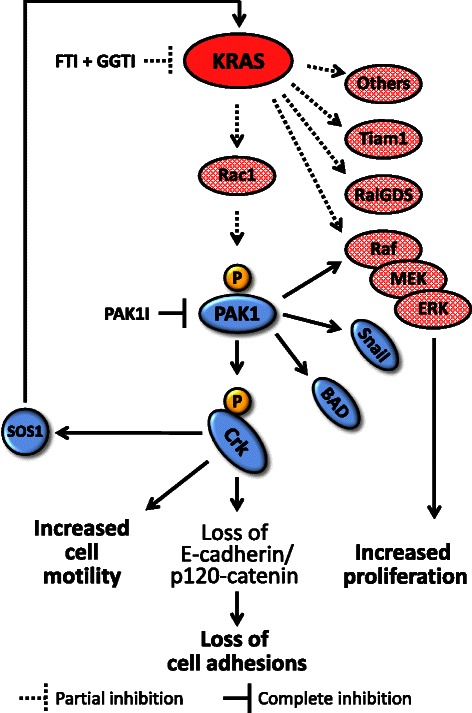
Cooperative disruption of signaling network downstream of mutated KRAS. Schematic view of KRAS/PAK1/Crk signaling pathway is demonstrated. Partial inhibition of KRAS signaling output by tolerable dose of prenylation inhibitors (i.e., FTI + GGTI) in addition to inhibition of a prominent KRAS effector (i.e., PAK1) result in dramatic anti-tumor effects. FTI: farnesyltransferase inhibitor, GGTI: geranylgeranyltransferase inhibitor, PAK1I: p21 associated kinase 1 inhibitor.

Considering inhibition of multiple downstream pathways might not be easily achievable, we reasoned that blockade of a prominent downstream pathway can be enhanced by addition of widespread partial blockade of KRAS signals. Therefore, we chose to utilize a PAK1 inhibitor [an inhibitor that blocks one of the prominent KRAS downstream pathways] and then add (FTI + GGTI) [which provide a partial signal blockade in all downstream KRAS effectors] for enhancing signal interruption of KRAS. As shown in Figures 7 and 9, this combination remarkably altered morphology, motility and proliferation of NSCLC cells. It appears that combination of prenylation inhibitors and PAK1 inhibitor adequately disrupt the signaling network downstream of KRAS (Figure 10) and provide a prominent anti-tumor effect. It is noteworthy that the observed synergy between prenylation inhibitors and a PAK1 inhibitor will provide the opportunity to use a low concentration of each inhibitor for blocking KRAS signals and thereby will enable us to avoid the risk of cellular toxicity.

## Conclusion

Recent evidence has emphasized on the role of PAK1 in transduction of KRAS oncogenic signal [1-4]. Our data here further support this role as we observed higher PAK1 activation in *KRAS* mutant as opposed to *KRAS* wild type clinical specimens. Furthermore, we provide data that proto-oncogene Crk is operative downstream of KRAS and the link from KRAS to Crk seems to be via PAK1. In summary, presented data introduce KRAS/PAK1/Crk axis (Figure 10) as one of the prominent signaling pathways downstream of mutated KRAS in lung cancer. In addition, we provide evidence that interruption of oncogenic KRAS signal is achievable by addition of Ras prenylation inhibitors to the inhibitors of prominent KRAS effectors. These findings also support the notion that inhibition of multiple KRAS effectors are required to achieve a noticeable biological effect.

## Additional files

Additional file 1: ***KRAS*****mutant NSCLC specimens express higher p-PAK1 compared to*****KRAS*****wild type specimens.**Box plots demonstrating distribution of p-PAK1 and E-cadherin expression measured by IHC in NSCLC specimens.
Additional file 2: **Proliferation rate of A549 and H157 cells following exposure to IPA-3, prenylation inhibitors or combination.** Line charts demonstrating the mean cell count of A549 and H157 cells following exposure to IPA-3 (5 μM); F: farnesyltransferase inhibitor; G: geranylgeranyltransferase inhibitor (500 nM each) or combination.

## Abbreviations

NSCLC: Non-small cell lung cancer
FTI: Farnesyltransferase inhibitor
GGTI: Geranylgeranyltransferase inhibitor
GGTase-I: Geranylgeranyltransferase type I
PAK1: p21 activated kinase 1
IPA-3: PAK1 inhibitor
