# Supplementary figures and images for: Significance of KRAS/PAK1/Crk pathway in non-small cell lung cancer oncogenesis
Source: BMC Cancer. 2015 May 9;15:381. doi: 10.1186/s12885-015-1360-4 (PMC4477307; doi:10.1186/s12885-015-1360-4)

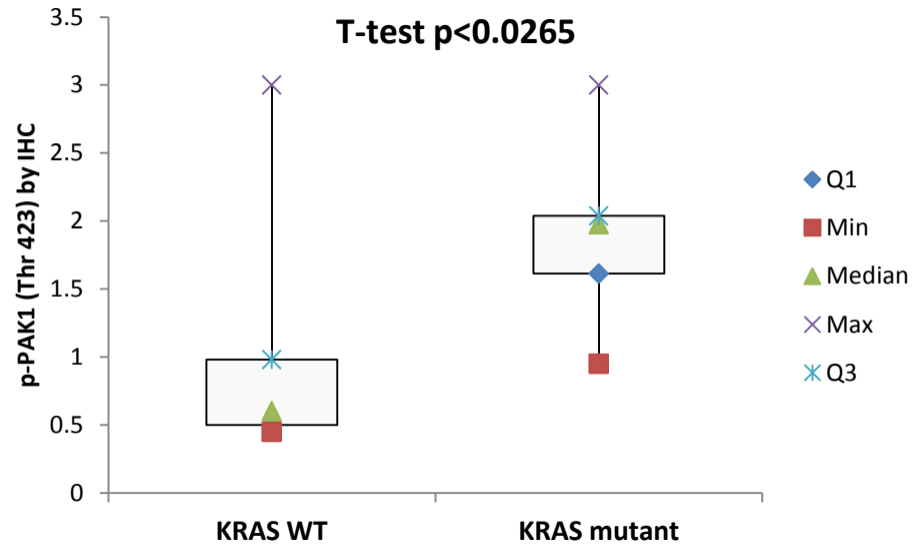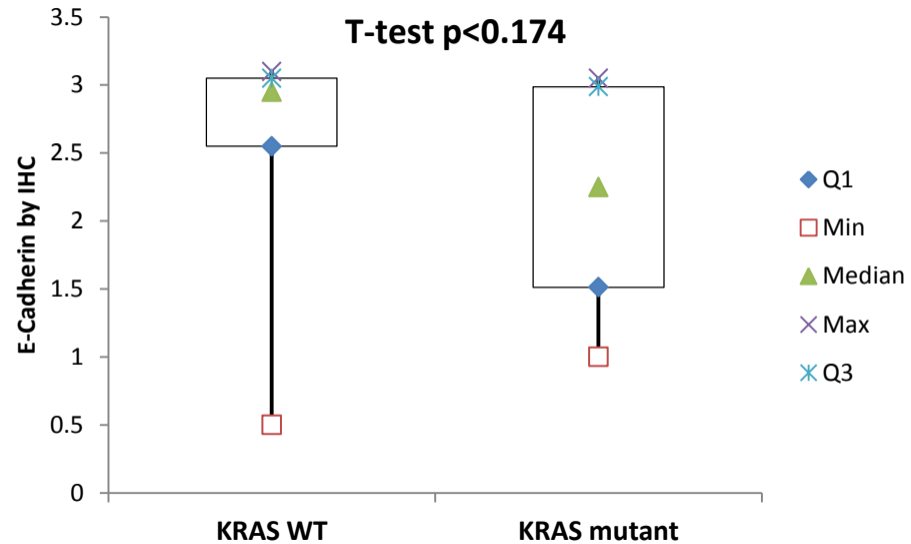

Supplement: Additional file 1: — KRASmutant NSCLC specimens express higher p-PAK1 compared toKRASwild type specimens.Box plots demonstrating distribution of p-PAK1 and E-cadherin expression measured by IHC in NSCLC specimens. [file 12885_2015_1360_MOESM1_ESM.pdf]

# H157

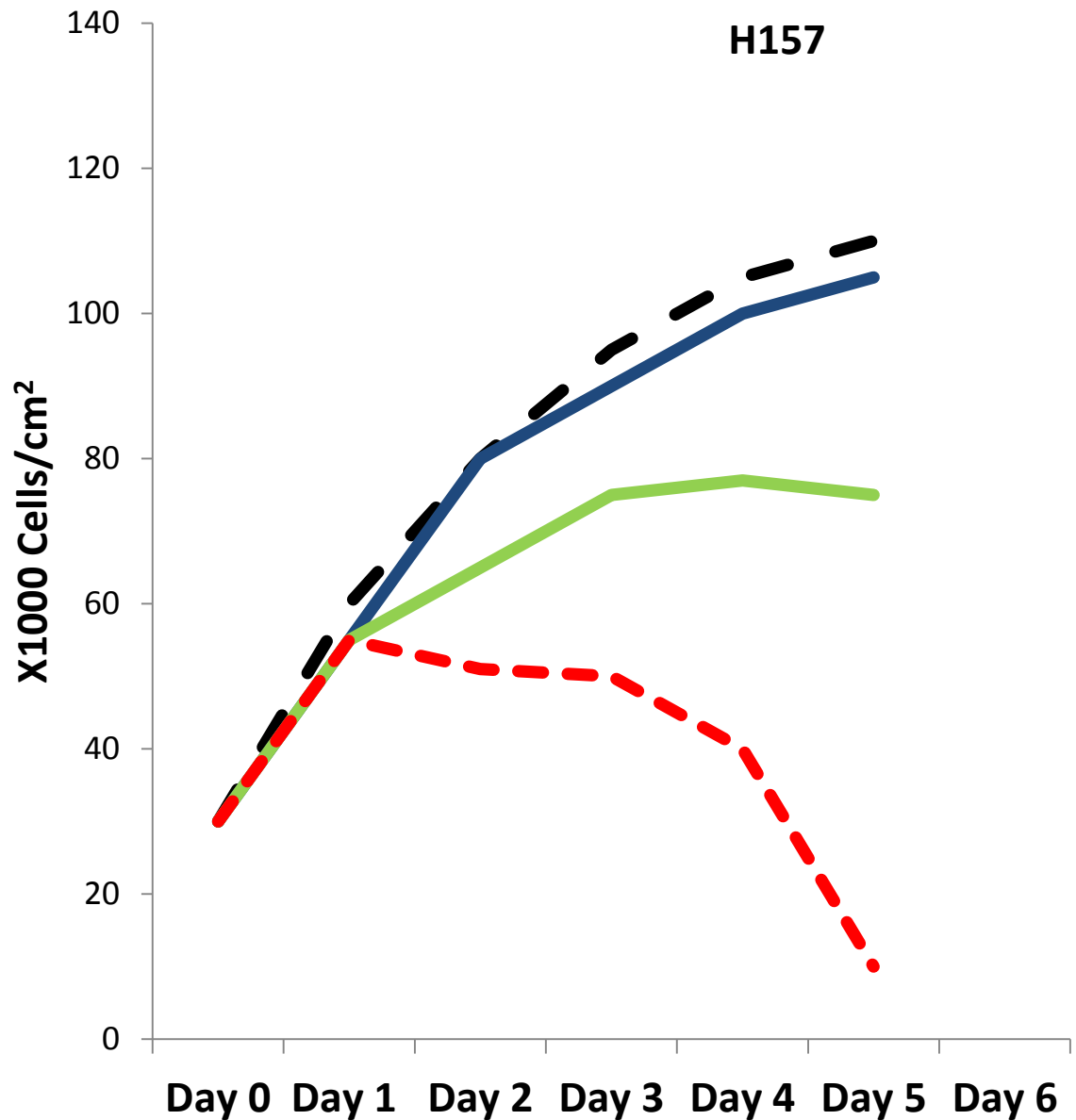

# A549

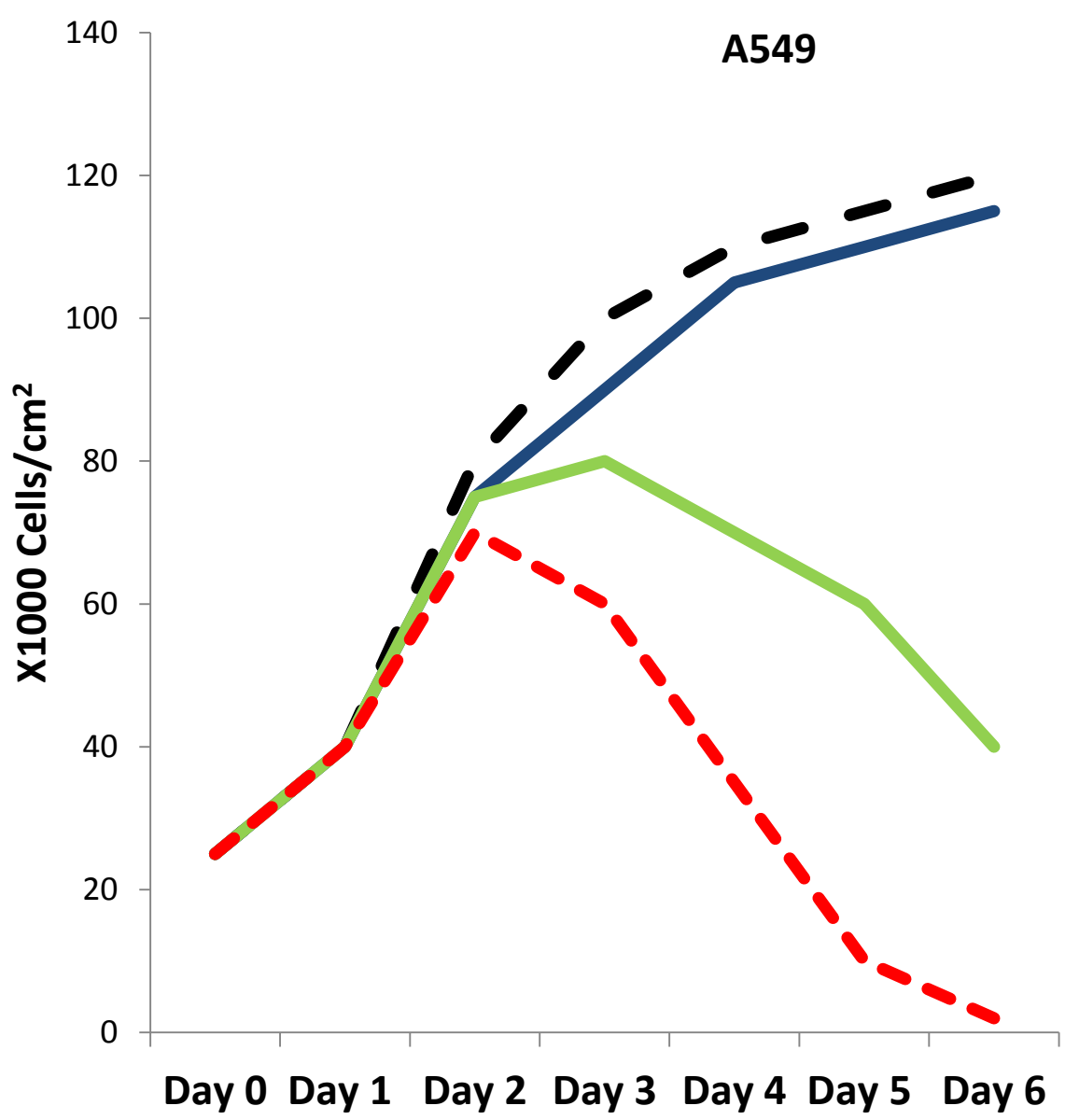

Supplement: Additional file 2: — Proliferation rate of A549 and H157 cells following exposure to IPA-3, prenylation inhibitors or combination. Line charts demonstrating the mean cell count of A549 and H157 cells following exposure to IPA-3 (5 μM); F: farnesyltransferase inhibitor; G: geranylgeranyltransferase inhibitor (500 nM each) or combination. [file 12885_2015_1360_MOESM2_ESM.pdf]
